# High resolution spectroscopy and chemical shift imaging of hyperpolarized ^129^Xe dissolved in the human brain in vivo at 1.5 tesla

**DOI:** 10.1002/mrm.26241

**Published:** 2016-04-15

**Authors:** Madhwesha Rao, Neil J. Stewart, Graham Norquay, Paul D. Griffiths, Jim M. Wild

**Affiliations:** ^1^ Academic Unit of Radiology, University of SheffieldSheffieldUnited Kingdom

**Keywords:** hyperpolarized gas, xenon spectroscopy, chemical shift imaging, human brain spectroscopy

## Abstract

**Purpose:**

Upon inhalation, xenon diffuses into the bloodstream and is transported to the brain, where it dissolves in various compartments of the brain. Although up to five chemically distinct peaks have been previously observed in ^129^Xe rat head spectra, to date only three peaks have been reported in the human head. This study demonstrates high resolution spectroscopy and chemical shift imaging (CSI) of ^129^Xe dissolved in the human head at 1.5 Tesla.

**Methods:**

A ^129^Xe radiofrequency coil was built in‐house and ^129^Xe gas was polarized using spin‐exchange optical pumping. Following the inhalation of ^129^Xe gas, NMR spectroscopy was performed with spectral resolution of 0.033 ppm. Two‐dimensional CSI in all three anatomical planes was performed with spectral resolution of 2.1 ppm and voxel size 20 mm × 20 mm.

**Results:**

Spectra of hyperpolarized ^129^Xe dissolved in the human head showed five distinct peaks at 188 ppm, 192 ppm, 196 ppm, 200 ppm, and 217 ppm. Assignment of these peaks was consistent with earlier studies.

**Conclusion:**

High resolution spectroscopy and CSI of hyperpolarized ^129^Xe dissolved in the human head has been demonstrated. For the first time, five distinct NMR peaks have been observed in ^129^Xe spectra from the human head in vivo. Magn Reson Med 75:2227–2234, 2016. © 2016 The Authors Magnetic Resonance in Medicine published by Wiley Periodicals, Inc. on behalf of International Society for Magnetic Resonance in Medicine. This is an open access article under the terms of the Creative Commons Attribution License, which permits use, distribution and reproduction in any medium, provided the original work is properly cited.

## INTRODUCTION

Hyperpolarized (HP) ^129^Xe MRI has gained interest ever since the first image was acquired from a biological sample 1. Xenon, when inhaled into the lungs, dissolves into the blood by diffusive gas exchange across the alveolar–capillary membrane 2, 3, 4, 5, 6. The fraction that dissolves in the blood is determined by the xenon blood–gas partition coefficient 7, 8, ratio of ventilated airspace volume to pulmonary blood volume 9, capillary perfusion and alveolar surface‐area‐to‐volume ratio. In the bloodstream, dissolved HP ^129^Xe is transported to distal organs by the systemic blood circulation. The longitudinal relaxation time $T_{1}$ of HP ^129^Xe in the human blood is sufficiently long (6 s to 8 s for arterial‐oxygenated blood and 3 s to 4 s for venous‐deoxygenated blood) for it to be detected in the distal organs 10, 11, 12, 13.

Recently, studies involving NMR spectroscopy and chemical shift imaging (CSI) of HP ^129^Xe dissolved in the brain have been conducted in rats 14, 15, 16, 17 and in humans 4, 18. Kershaw et al and Nakamura et al 19, 20 observed five peaks in ^129^Xe spectra from the rat head, which they attributed to jaw muscle (187–191 ppm), white matter (191–194 ppm), gray matter (193–197 ppm), fat tissue outside the brain (197–201 ppm), and red blood cells (210 ppm). The feasibility of imaging rat brain using HP ^129^Xe CSI was demonstrated by Swanson et al 16. Using HP ^129^Xe, Mazzanti et al 14 explored the possibility of detecting increased brain perfusion and functional activity following sensory stimulation in rats and Zhou et al 15 demonstrated the detection of ischemic stroke in rats. To our understanding, the only two studies of HP ^129^Xe in the human head to date are from Mugler et al 4 and Kilian et al 18, 21 who presented time resolved spectra depicting the uptake of HP ^129^Xe into the human brain in vivo. Subsequently, Kilian et al 18, 21 reported preliminary results of HP ^129^Xe CSI in the human brain at 3.0 Tesla (T) and estimated $T_{1}$ relaxation of HP ^129^Xe dissolved in gray matter and white matter to be 14 s and 8 s, respectively.

Unlike the HP ^129^Xe studies conducted in rats 19, 20, the preliminary human studies have revealed two dissolved peaks which were attributed to gray matter (196 ppm) and white matter (193 ppm) 18, 21, and one report showed evidence of a third peak at 186 ppm 21. Thus, the motivation of our study at 1.5T was to perform (i) high resolution ^129^Xe spectroscopy of the human head in vivo and (ii) CSI to spatially resolve the compartments of the brain responsible for the peaks observed.

## METHODS

### RF Coil and Calibration

**Figure 1:**
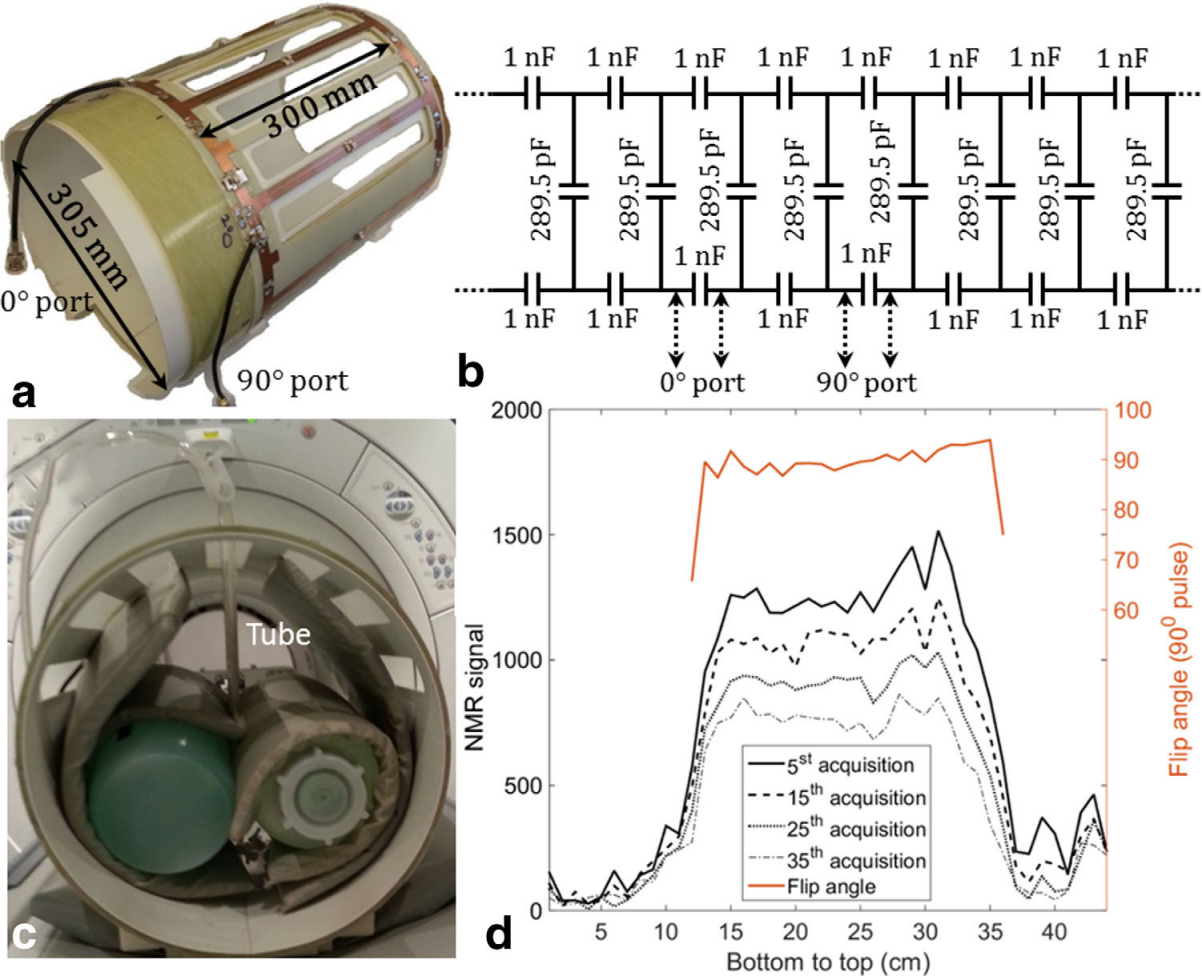
RF birdcage coil for ^129^Xe brain MR spectroscopy and chemical shift imaging. **a**: Photograph of the RF birdcage coil. **b**: Schematic of the RF birdcage coil. **c**: Picture of the experimental setup for calculation of flip angle profile. **d**: ^129^Xe NMR signal measured after the 5^th^, 15^th^, 25^th^, and 35^th^ RF pulses (black lines) indicating depletion in polarization and the flip angle profile for a 90° RF pulse (red line).

For the construction of a custom ^129^Xe radiofrequency (RF) coil for the human head, a rigid mechanical former was recycled from a disused ^1^H head birdcage coil. An eight‐leg birdcage coil of band pass topology was constructed with a capacitance of 289.5 pF (270 pF + 18 pF + 1.5 pF) and 1000 pF on the legs and on the end ring, respectively, as shown in Figure 1b. A photograph of the RF birdcage coil is shown in Figure 1a. The end ring diameter was 305 mm and the leg length was 300 mm as shown in Figure 1a. The capacitors used were of 10C package (Dalian Dalicap Technology Co., Ltd, Dalian, China). The birdcage coil was driven in quadrature transmit–receive mode. RF measurements were performed using an Agilent 5061B Network Analyzer (Keysight Technologies, Santa Rosa, CA).

The RF power required to achieve a desired flip angle was calculated based on a standard calibration procedure, whereby the rate of depletion of polarization of HP ^129^Xe resulting from repeated acquisitions using hard RF pulse within a short time‐interval ( $TR\ll \;T_{1}$) was fit to ${\;cos}^{n‐1}\alpha$, where n is the number of repeated acquisitions and α is the flip angle for the transmit RF power. To calculate the variation in flip angle across the coil, a plastic tube with 6 mm inner diameter was filled with HP ^129^Xe and positioned radially within the RF coil as shown in the photograph in Figure 1c, and the coil was loaded with a standard nickel chloride (NiCl_2_·6H_2_0:H_2_0) phantoms. The length of the tube was 36 cm, which covered the diameter of the RF coil.

Using a fast spoiled gradient echo sequence with a soft (Gaussian) RF pulse, with frequency encoding applied only along one direction (anterior to posterior as shown in Figure 1c), the flip angle profile along the diameter of the RF coil was calculated by measuring the depletion of polarization of the HP ^129^Xe in each voxel along the length of the tube as shown in Figure 1d. Acquisition parameters were: echo time (TE) = 3.6 ms, repetition time (TR) = 18.9 ms, matrix = 1 (phase) × 44 (frequency), slice thickness = 200 mm, number of slices = 1, field of view (FOV) = 44 cm, RF pulse duration = 800 µs, and the acquisition lasted 800 ms. A total of 39 repeated acquisitions were performed. $T_{1}$ relaxation and gas diffusion were both neglected for the calculation.

### MRI Methods

All in vivo NMR with ^129^Xe experiments were performed with approval from the UK National Research Ethics Committee under a protocol for the evaluation of novel MRI methods for imaging hyperpolarized xenon in the lungs, brain, and heart of healthy human volunteers. MRI experiments were performed on a GE whole‐body 1.5T Signa® HDx scanner. All in vivo experiments were conducted following inhalation of isotopically enriched xenon (87% ^129^Xe) from 1 L Tedlar® bags. ^129^Xe gas was polarized using spin exchange optical pumping to around 25% polarization 22. Chemical shift values were calibrated with respect to the resonance of ^129^Xe in the gas‐phase measured in a Tedlar® bag positioned at the isocenter of the scanner. In vivo experiments were conducted on three healthy male volunteers with the mean age of 29 years and standard deviation of 3 years.

### Wideband Spectroscopy

The xenon gas dose for the acquisition was 1 L. The acquisition was performed under breath‐hold commencing immediately after the inhalation of the gas and lasted for 20 s thereafter. After inhalation, the residual gas in the Tedlar® bag was placed on the neck of the subject to obtain a reference HP ^129^Xe gas‐phase signal. A total of 20 discrete free induction decays (FIDs) were acquired with a flip angle of 20° and TR of 1 s. The transmit RF pulse was a hard pulse with a pulse duration of 500 µs. The center frequency was set to 197.93 ppm downfield (17,660,800 Hz) from the ^129^Xe gas‐phase resonance and the receiver bandwidth was 674.16 ppm (11.9 kHz). The spectral resolution was 0.16 ppm (2.9 Hz) with 4096 sampling points in the spectrum. All 20 acquired complex spectra were averaged to improve the signal‐to‐noise ratio (SNR). No spectral line broadening filters were applied.

### High Resolution Spectroscopy

For high resolution spectroscopy, data acquisition was performed during a 20 s breath‐hold, commencing 4 s after inhalation of a 1 L dose of xenon gas. Ten discrete FIDs were acquired with a TR of 2 s and a flip angle of 45°. The transmit RF pulse was a hard pulse with a pulse duration of 500 µs. The center frequency was set to 197.93 ppm downfield (17,660,800 Hz) from the ^129^Xe gas‐phase resonance and the receiver bandwidth was 136.09 ppm (2403 Hz). The spectral resolution was 0.033 ppm (0.58 Hz) with 4096 sampling points in the spectrum. All 10 acquired complex spectra were averaged and no spectral line broadening filters were applied.

### Chemical Shift Imaging

To spatially localize the ^129^Xe spectral peaks anatomically in the human brain, 2D CSI was conducted by applying phase‐encoding in the anterior–posterior and right–left directions for axial‐plane CSI, anterior–posterior and superior–inferior directions for sagittal‐plane CSI, and right–left and superior–inferior directions for coronal‐plane CSI. FIDs were acquired for each phase‐encoding step using a pulse‐acquire sequence without slice selection. The transmit RF pulse was as described earlier. The center frequency was set to 197.93 ppm downfield (17,660,800 Hz) from the ^129^Xe gas‐phase resonance and the receiver bandwidth was 136.05 ppm (2403 Hz). The spectral resolution was 2.1 ppm (37 Hz) with 64 sampling points per spectrum. The flip angle was 10°, and the TR was 45 ms. For a matrix size of 12 × 12 and a FOV of 24 cm, 144 phase‐encoded spectra were acquired with an effective in‐plane spatial resolution of 20 mm × 20 mm. The acquisition was performed under breath‐hold, commencing 16 s after the inhalation of a 1 L dose of xenon gas and lasted for 6.5 s thereafter. No spectral line broadening filters were applied.

For anatomical reference, ^1^H MR images were acquired (in each of the three anatomical planes) using an inversion recovery gradient echo pulse sequence in a separate scan with the same FOV as the ^129^Xe CSI acquisition. The imaging parameters were: TE = 5.2 ms, inversion time = 450 ms, TR = 12.4 ms, flip angle = 20°, bandwidth = 15.6 kHz, slice thickness = 1.6 mm and matrix size = 512 × 512. For the purpose of assignment of a particular resonance to a particular brain compartment, the ^129^Xe chemical shift images were superimposed on the corresponding anatomical reference ^1^H images. Each pixel of the HP ^129^Xe CSI represents the maximum value of a particular chemical shift peak at that spatial location. An image intensity threshold determined by visual inspection was applied to the 2D CSI images of dissolved HP ^129^Xe to eliminate background noise. The threshold for each of the 2D CSI images were determined and applied individually.

## RESULTS

### RF Coil and Calibration

The quality factor (Q) of the birdcage RF coil in the unloaded condition was 270 and in the loaded condition was 86. The ratio of the Q factor in unloaded to loaded condition was, therefore, 3.1. The isolation (transfer coefficient, ${\;S}_{ij,\;(i\neq j)}$) between the in‐phase and the quadrature‐phase ports of the RF birdcage coil was lower than −15 dB. Figure 1d shows the measured ^129^Xe NMR signal after the 5^th^, 15^th^, 25^th^, and 35^th^ RF pulse with frequency encoding applied only along one direction (anterior–posterior), indicating depletion of polarization. For a nominal 90° RF pulse, the standard deviation of the flip angle measured across the diameter of the coil was 5.9° (6.56%) and the flip angle profile is shown in Figure 1d.

### Spectroscopy

**Figure 2:**
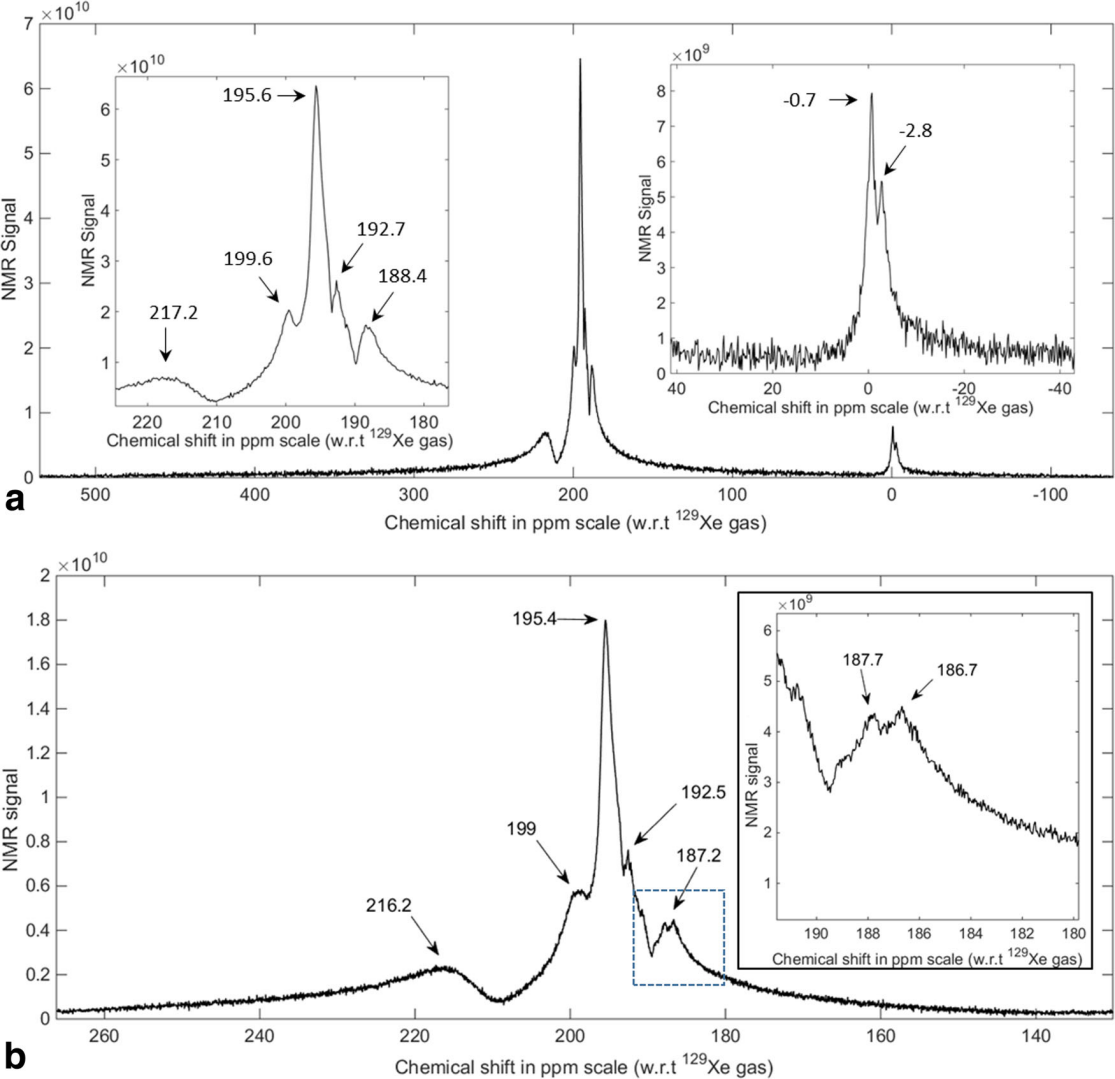
In vivo magnitude spectra of HP ^129^Xe dissolved in the human head. **a**: Spectrum with wide bandwidth (674.16 ppm) and a spectral resolution of 0.16 ppm. **b**: Spectrum with a narrow bandwidth (136.09 ppm) and a higher spectral resolution of 0.033 ppm. Note: (a) and (b) represent two separate acquisitions. Subject: male 31 years old.

An averaged spectrum of HP ^129^Xe dissolved in the human head acquired with a wide receiver bandwidth is shown in Figure 2a. The spectrum exhibits two distinct peaks near 0 ppm assigned to ^129^Xe in the gaseous phase. Near the center frequency of the spectrum, five distinct dissolved ^129^Xe peaks from the human head can be observed, at 188.4 ppm, 192.7 ppm, 195.6 ppm, 199.6 ppm, and 217.2 ppm.

An averaged whole head spectrum acquired from HP ^129^Xe dissolved in the human head with high spectral resolution (0.033 ppm) is shown in Figure 2b. The spectrum exhibits five distinct peaks: 187.2 ppm, 192.5 ppm, 195.4 ppm, 199.0 ppm, and 216.2 ppm.

### Chemical Shift Imaging

**Figure 3:**
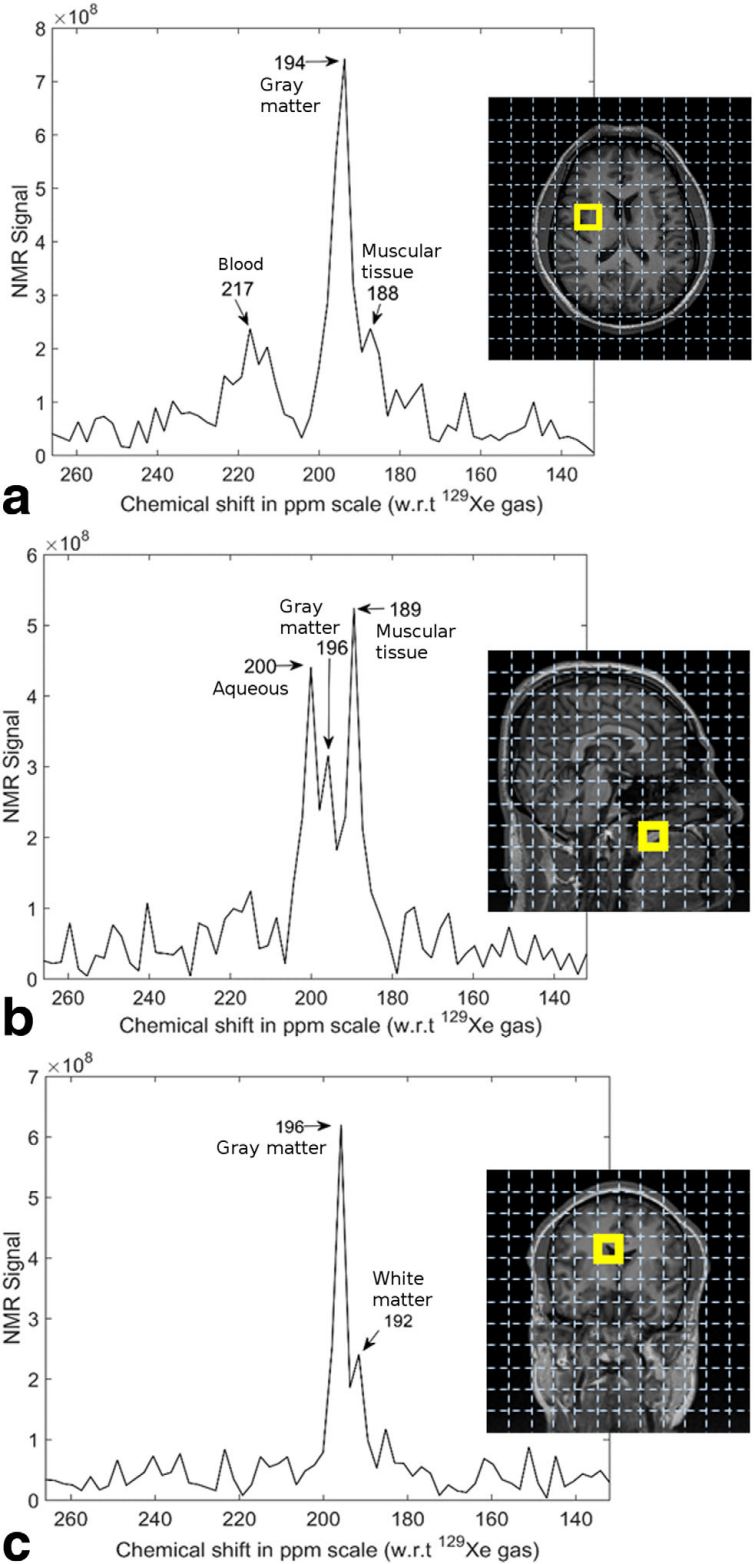
Individual spectra acquired in a 2D chemical shift imaging experiment at arbitrary locations in the head. **a**: Axial plane spectrum from the left center of the head, showing peaks at 188 ppm, 194 ppm, and 217 ppm. **b**: Sagittal plane spectrum from the anterior inferior part of the head, showing peaks at 189 ppm, 196 ppm, and 200 ppm. **c**: Coronal plane spectrum from the superior center of the head, showing peaks at 192 ppm and 196 ppm. The peaks have been labeled to indicate the compartment of the head with which they are assigned (see the Discussion section). Subject: male 31 years old.

**Figure 4:**
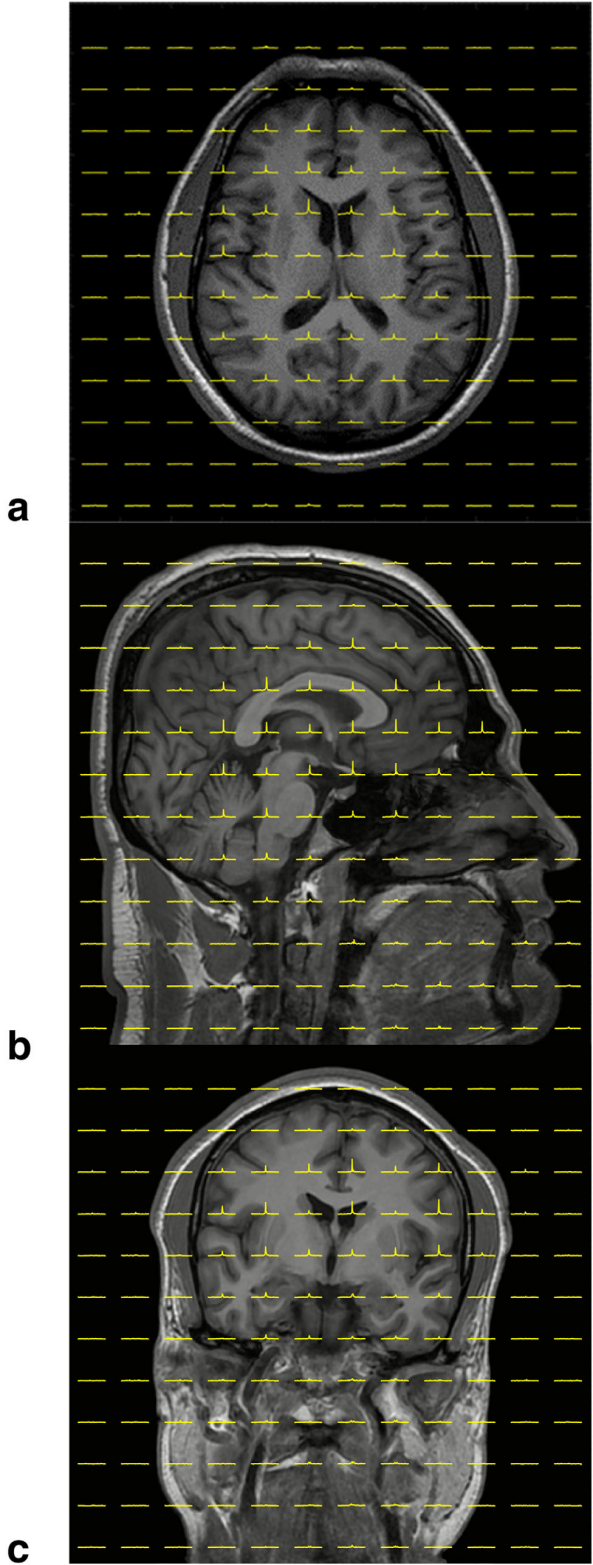
Spatially resolved ^129^Xe spectra of the head in all the three imaging planes superimposed on corresponding anatomical ^1^H images. **a**: Axial plane. **B**: Sagittal plane. **C**: Coronal plane. Subject: male 31 years old.

To demonstrate that the SNR of spatially resolved spectra from the CSI experiment of the human head are sufficient, example spectra (at arbitrary locations) for each of the anatomical planes are shown in Figure 3. It can be observed, that the SNR of these spatially resolved spectra are sufficient to enable distinction of the peaks at 188 ppm, 192 ppm, 196 ppm, 200 ppm, and 217 ppm, and that the signal amplitudes are clearly prominent above the noise floor. Spatially resolved spectra in all the three imaging planes superimposed on corresponding anatomical ^1^H images are shown in Figure 4.

**Figure 5:**
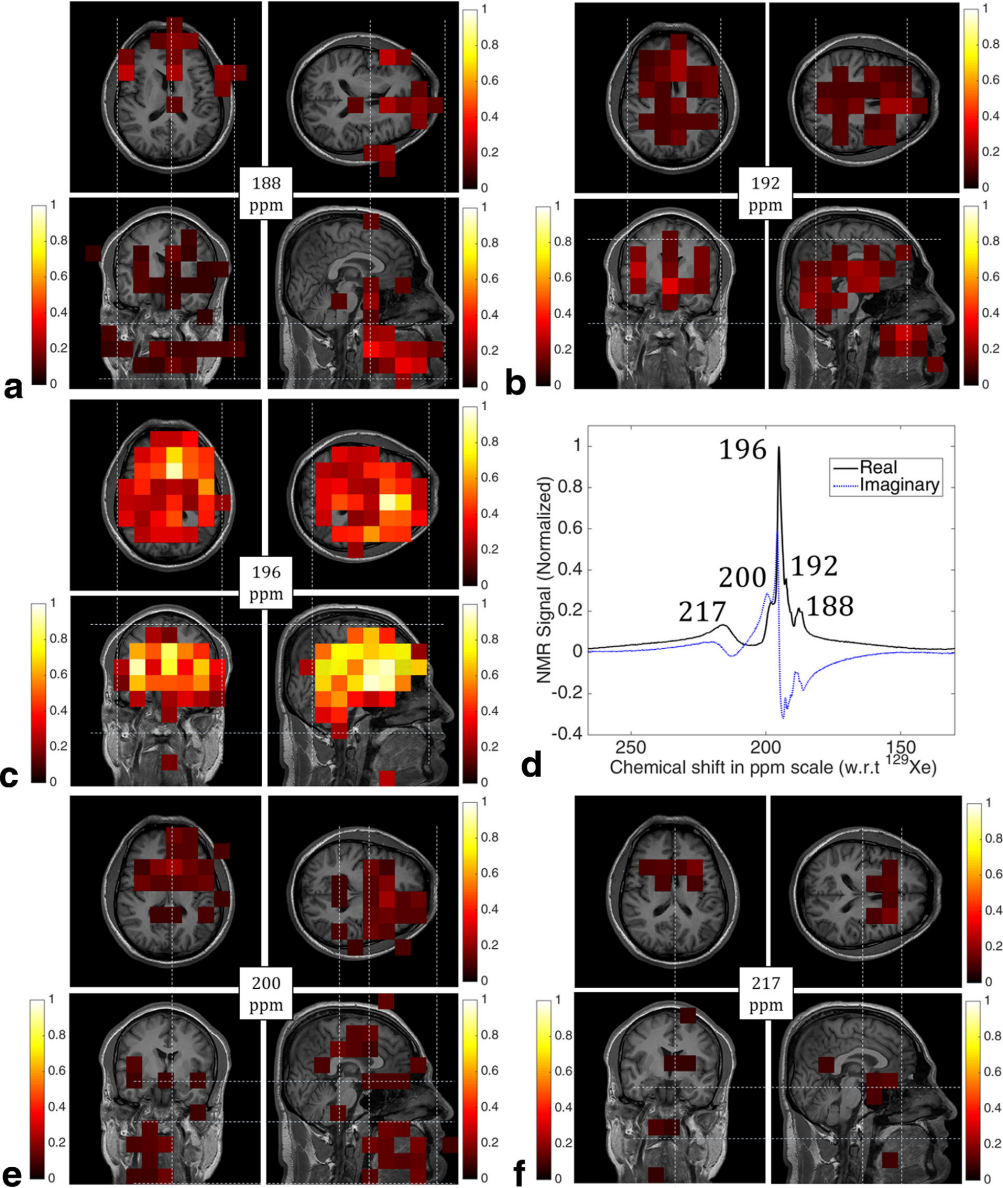
The 2D chemical shift images of spatially resolved peaks from ^129^Xe in the human head, superimposed (in color) on ^1^H reference images (gray scale) in all the three anatomical planes. **a**: 188 ppm ‐ tissue in the cheek muscle and the midbrain/brainstem. **b**: 192 ppm ‐ white matter and cartilaginous soft‐tissue. **c**: 196 ppm ‐ gray matter. **e**: 200 ppm ‐ body interstitial fluid/plasma, fat tissue outside the brain and cerebrospinal fluid. **f**: 217 ppm ‐ red blood cells. The threshold for each of the 2D CSI images were determined and applied individually. Color bars represent arbitrary units normalized to the maximum NMR signal intensity of hyperpolarized ^129^Xe dissolved in the gray matter. **d**: Phase‐corrected real and imaginary parts of the spectrum from Figure 2b, showing HP ^129^Xe dissolved in the human head in vivo with a spectral resolution of 0.033 ppm and a bandwidth of 136.09 ppm. Subject: male 31 years old.

Chemical shift images corresponding to ^129^Xe NMR peak from the head at 188 ppm, 192 ppm, 196 ppm, 200 ppm, and 217 ppm, superimposed (in color) on corresponding anatomical ^1^H images are shown in Figures 5a–c,e,f. Figure 5d shows the spectrum from Figure 2b split into its constituent real and imaginary parts after zeroth order phase‐correction using the 195.4 ppm (gray matter) peak as a reference. The full width half maximum of the peak at 195.4 ppm (gray matter) in Figure 5d is 36 Hz.

## DISCUSSION

In Figure 2a, the peak at −0.7 ppm can be attributed to residual gas in the Tedlar® bag placed on the subject's neck. The ^129^Xe gas‐phase resonance being shifted by −0.7 ppm (instead of 0 ppm) suggests $B_{0}$ variation of 1 μT due to the Tedlar® bag not being positioned at the isocenter of the magnet combined with bulk susceptibility shifts related to the subject's head. Similarly, we postulate that the peak at −2.8 ppm is related to detection of ^129^Xe gas in the smaller airways of the lungs in the stray field of the RF coil. The up‐field (negative) chemical shift of this ^129^Xe gas in the smaller airways of the lungs could be due to a combination of effects, including; bulk magnetic susceptibility shift effects 5, the chemical shift associated with mixing with oxygen 23 and the fact that while an average adult head is positioned at the isocenter, the lungs lie further away from the isocenter at a lower static magnetic field strength than the isocenter and would experience a lower Larmor frequency.

The chemical shift values reported in Figure 3 are marginally different from the values reported in Figure 2, most likely due to the considerable difference in spectral resolution between the two acquisitions. In addition, it can be observed that some of the five peaks (from Figure 2) are absent in some of the spectra in Figure 3. This indicates that the particular biochemical compartment giving rise to a specific chemical shift value is not present at that particular voxel location.

From the anatomical location of the ^129^Xe NMR peaks in the chemical shift images superimposed on corresponding ^1^H images in Figure 5, we propose the assignment of each individual peak to a particular compartment of the head as follows:

### 188 ppm

From Figure 5a, the peak at 188 ppm appears to be most intense in the anterior region of the axial plane image. The sagittal plane image indicates that most of the signal from this peak arises from regions below the eyes, above the jawline and between the nose and ears. In the coronal plane image, it is evident that the signal intensity is approximately symmetric on both right and left sides of the head. In addition, this image shows that some contribution arises from a location at the midbrain. We allocate the peak at 188 ppm to HP ^129^Xe dissolved in soft muscular tissue in cheek and midbrain.

### 192 ppm

From Figure 5b, it can be observed that the peak at 192 ppm appears to predominantly arise from ^129^Xe dissolved in the brain. In the axial plane, the peak at 192 ppm is most intense in the brain with some evidence of tracts. Similarly, in the coronal plane image, the peak at 192 ppm appears to be most intense in the central brain, again with some evidence of tracts. In the sagittal plane image, a contribution to the 192 ppm peak can be observed from ^129^Xe in the region of the nasal cavity. Thus, it can be postulated that the peak at 192 ppm corresponds to HP ^129^Xe dissolved in white matter (Commissural and Projection tracts) and cartilaginous soft‐tissue.

### 196 ppm

Figure 5c shows that the ^129^Xe peak at 196 ppm is predominantly localized to the brain; it can be clearly seen in images from the all three anatomical planes that the peak not only emanates from the brain, but does not appear anywhere else. Thus it can be concluded that the peak at 196 ppm corresponds to HP ^129^Xe dissolved in the gray matter.

Unlike the case of xenon gas in an isolated bag, in the human brain in vivo, ^129^Xe will undergo diffusive chemical exchange with other compartments of the brain that come in to contact with each other (such as cerebral blood, interstitial fluid, and cerebrospinal fluid). Existence of any such chemical exchange will broaden the line‐width of the NMR peaks of the compartments under exchange. Nevertheless, under these conditions the full width half maximum of a particular spectral peak will provide a lower limit estimate of the ${\;T}_{2}^{*}$ relaxation time from a given voxel. The full width half maximum of 36 Hz for the gray matter peak in Figure 5d corresponds to ${\;T}_{2}^{*}\geq$ 8.8 ms.

### 200 ppm

From Figure 5e, the peak at 200 ppm appears to originate from ^129^Xe located in the anterior region of the head, as seen from the axial plane image. In the sagittal plane image, most of the signal appears to be from regions among the eyes, nose, and ears, and extends below toward the jaw. In the coronal plane image, the signal appears to arise from the center of the head in the right–left direction. In all the three anatomical planes, the signal appears to coincide with the location of the ventricles. Thus, the peak at 200 ppm is likely from HP ^129^Xe dissolved in body interstitial fluid/plasma, fat tissue outside the brain, and cerebrospinal fluid.

### 217 ppm

From Figure 5f, in all three anatomical planes, the only location where the peak at 217 ppm shows considerable signal intensity is at the location of the Circle of Willis. This peak at 217 ppm can, therefore, be allocated to HP ^129^Xe dissolved in the red blood cells. It is worth noting that this peak is broader than the others, most probably due to the diffusive chemical exchange between ^129^Xe dissolved in various compartments, and to the fact that blood oxygenation will vary across the head, resulting in a dispersion of the chemical shift due to difference in the oxygenation state of the hemoglobin 24.

The assignment of the chemical shift values with the particular biochemical environments as described above is supported by earlier studies, which include in vivo reports of ^129^Xe in the rat and human head and in vitro studies of ^129^Xe dissolved in human blood. These earlier studies suggested the following peak assignments: 187.2 ppm, muscle 19, 20, 21; 192.7 ppm, white matter (and soft‐tissue in this study) 18, 19, 20, 21; 195.6 ppm, gray matter 18, 19, 20, 21; 199.6 ppm, aqueous solution (cerebrospinal fluid, plasma, and interstitial fluid in this study) 10, 11, 24, 25, 26 and fat/lipid tissue outside the brain 19, 20; and 217.2 ppm, red blood cells 4, 10, 24, 26.

**Figure 6:**
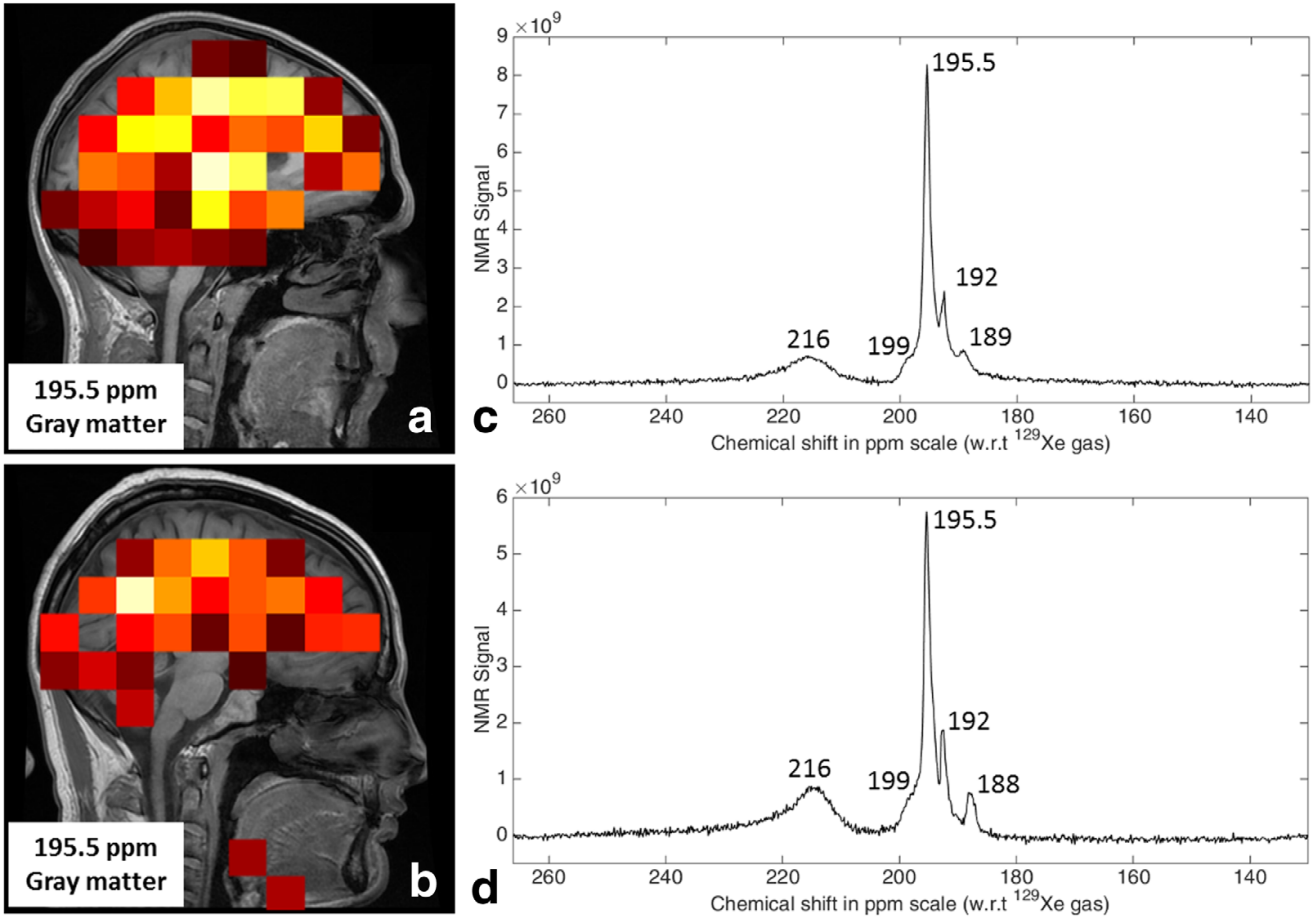
Chemical shift images of the spectral peak at 195.5 ppm assigned to ^129^Xe dissolved in gray matter: male subject 25 years old (**a**) and male subject 30 years old (**c**). Spectra (real part) of HP ^129^Xe dissolved in the human brain in vivo acquired with same acquisition parameter as high resolution spectrum: male subject 25 years old (**b**) and male subject 30 years old (**d**).

The peaks seen in whole brain spectroscopy and their anatomical distribution from CSI, were found to be consistent between the subjects as shown in Figure 6. The SNR of the obtained spectra was found to be somewhat variable between the subjects. As SNR is limited by the quantity and polarization of ^129^Xe delivered to the brain by the systemic blood circulation, any variability in the polarization of the SEOP process, gas dose inhaled or ${\;T}_{1}$ losses related to different lung‐to‐brain transit times will affect the observed SNR. Assessment of these factors, their variability and their effect on measured spectroscopic SNR of dissolved ^129^Xe in the brain is the subject of future work.

On closer inspection of Figure 2b, it can be seen that the peak at 187.2 ppm appears to be comprised of two closely spaced peaks at 186.7 ppm and 187.7 ppm which requires further investigation. In addition, a peak at 190.6 ppm can be observed in both Figure 2a inset and 2b which also requires further investigation in future work.

## CONCLUSIONS

This is the first time that five distinct ^129^Xe NMR peaks have been observed in the human head in vivo and we believe this to be the first report of ^129^Xe CSI in the human head in vivo at 1.5T. Although earlier studies evidenced the presence of 5 ^129^Xe NMR peaks in the rat head 19, 20, only two or three peaks have been previously detected from ^129^Xe in the human head from spectra of significantly lower SNR and spectral resolution than those in this study 4, 18. Our results should provide a useful reference for future ^129^Xe NMR spectroscopy studies of the human brain. Furthermore, the methods established here may be directly applied to quantify the dynamics of xenon uptake into the human brain and probe the potential pathophysiological changes induced by functional abnormalities.
